# Rural Subsistence Maize Farming in South Africa: Risk Assessment and Intervention models for Reduction of Exposure to Fumonisin Mycotoxins

**DOI:** 10.3390/toxins11060334

**Published:** 2019-06-12

**Authors:** Johanna Alberts, John Rheeder, Wentzel Gelderblom, Gordon Shephard, Hester-Mari Burger

**Affiliations:** Mycotoxicology Research Group, Institute of Biomedical and Microbial Biotechnology, Cape Peninsula University of Technology, Bellville 7535, South Africa; rheederjp@cput.ac.za (J.R.); gelderblomw@cput.ac.za (W.G.); gshephard@mweb.co.za (G.S.); burgerh@cput.ac.za (H.-M.B.)

**Keywords:** subsistence maize farming, fumonisin, exposure, risk assessment, reduction, intervention models

## Abstract

Maize is a staple crop in rural subsistence regions of southern Africa, is mainly produced for direct household consumption and is often contaminated with high levels of mycotoxins. Chronic exposure to mycotoxins is a risk factor for human diseases as it is implicated in the development of cancer, neural tube defects as well as stunting in children. Although authorities may set maximum levels, these regulations are not effective in subsistence farming communities. As maize is consumed in large quantities, exposure to mycotoxins will surpass safe levels even where the contamination levels are below the regulated maximum levels. It is clear that the lowering of exposure in these communities requires an integrated approach. Detailed understanding of agricultural practices, mycotoxin occurrence, climate change/weather patterns, human exposure and risk are warranted to guide adequate intervention programmes. Risk communication and creating awareness in affected communities are also critical. A range of biologically based products for control of mycotoxigenic fungi and mycotoxins in maize have been developed and commercialised. Application of these methods is limited due to a lack of infrastructure and resources. Other challenges regarding integration and sustainability of technological and community-based mycotoxin reduction strategies include (i) food security, and (ii) the traditional use of mouldy maize.

## 1. Introduction

Species of the fungal genera, *Aspergillus*, *Fusarium* and *Penicillium* are known to produce mycotoxins in a wide range of food commodities worldwide [1]. It is estimated that mycotoxins contaminate a quarter of the world’s food crops resulting in an annual economic loss of approximately US $1.4 billion in the United States alone [2]. Aflatoxin contamination of maize was estimated at US $75–100 million in 1985 [3], and recently between US $52.1 million and 1.68 billion annually in the United States [4]. When considering the worldwide regulation of mycotoxins in food, low to medium income countries seem to be affected the most. It was estimated that enforcement of strict regulations regarding aflatoxin contamination by the European Union would result in the rejection of 64% of imports of cereals, dried fruits and nuts from African countries with an estimated trade loss per year of approximately US $670 million [5]. In low income countries this can result in an increased risk of mycotoxin exposure with rural subsistence farming communities being the most vulnerable.

Mycotoxin contamination of crops has been associated with the development of a large range of chronic diseases including cancer, digestive, blood and nerve disorders in humans and/or animals [6]. The health impact of mycotoxin exposure, specifically in malnourished populations residing in low income countries, is therefore a concern. When considering the critical effects of food contaminants in conjunction with other risk factors the toxicity, carcinogenicity, immunotoxicity and oestrogenicity of mycotoxins play a major role in disease outcomes in low income countries. They add significantly to morbidity and it is estimated to account for 40% of lost disability adjusted life years [7,8]. This is of particular importance in rural areas where people are exposed to a cocktail of mycotoxins, some of which are far above the acceptable levels. The potential of synergistic interaction of mycotoxin exposure not only affects cancer development but also chronic and infectious diseases. Strategies to reduce mycotoxin exposure are therefore of major importance, especially in low income countries due to challenges regarding farming, inadequate storage of crops and monocereal dietary practices. The impact of climate change is likely to further deteriorate the situation which raises the need for strategic intervention models to mitigate mycotoxin exposure and the adverse health effects [4,9].

*Fusarium verticillioides* (previously known as *F. moniliforme*) has been characterised as the most prevalent fungus on maize, the major dietary staple in rural subsistence farming communities in South and southern Africa [10,11]. Apart from the induction of leukoencephalomalacia (LEM) in equines by naturally contaminated maize or maize fungal culture material, it caused diverse toxic lesions in experimental animals [12,13,14]. Of these the induction of liver cancer in rats suggests that carcinogenic principles are produced by the fungus [15,16]. Subsequent studies implicated the fungus in the development of human oesophageal cancer (OC) in the Transkei region of the Eastern Cape Province (EC), South Africa [17,18]. Similar etiological associations were reported with high OC as well as liver cancer incidence rates in China [19].

The characterisation of the fumonisin B (FB) mycotoxins opened new research directions to further investigate the role of the fungus in disease development in humans and animals. Detailed studies in animals indicated that fumonisin exhibited carcinogenic effects in rats and mice, while it was found to be the causative principle for the induction of LEM in horses and pulmonary oedema in pigs [20,21,22,23]. Recent studies also implicate the mycotoxin in the development of neural tube defects in humans following the finding that FB_1_ induces neural tube defects in mice [24,25]. After the development of sensitive analytical techniques for their detection in food commodities [25], analytical results indicated that they are implicated in the development of OC, liver cancer and neural tube defects [26,27,28,29,30]. These investigations were further extended by utilising plasma and urinary biomarkers of exposure to more accurately assess exposure in human populations and assessing the risk of the development of OC, neural tube defects and growth retardation [31].

From a regulatory perspective, the toxicological effects in rats and mice [32] were instrumental in defining the provisional maximum tolerable daily intake (PMTDI) of 2 µg kg^−1^ body weight (bw) day^−1^ set by the Joint Food and Agriculture Organization of the United Nations (FAO)/World Health Organization (WHO) Expert Committee on Food Additives (JECFA) [33]. Subsequently the International Agency for Research on Cancer (IARC) characterised the fumonisins as a Group 2B carcinogen, i.e., a possible carcinogen to humans [34]. Based on these risk assessments, various maximum levels (MLs) in maize aimed at governing international trade have been implemented by different countries. The Codex Alimentarius Commission (CAC) has set MLs in whole maize and maize meal at 4 and 2 mg/kg, respectively, which were recently adopted by the South African National Department of Health [35]. These ML’s are not sufficient to protect rural subsistent maize farming communities against the adverse effects of the fumonisins as the maize consumption profiles resulted in probable daily intake (PDI) levels far above the PMTDI [36,37,38]. To safeguard these rural subsistent farming communities, studies are focusing on reduction of FB in food utilising different chemical and biological reduction and/or decontamination approaches. The current paper will address these aspects in more detail in order to focus on the effect of the mycotoxin regulations in low income countries, such as South Africa, and devise applicable intervention models to reduce exposure with subsequent validation of the efficacy utilising sensitive biomarkers of exposure.

## 2. Subsistence Maize Farming and Agricultural Practices in South Africa

Foodstuffs such as sorghum, millet, cassava and peanuts are important staples and sources of revenue in the rural subsistence regions of southern Africa, but are prone to mycotoxin contamination [39,40,41,42,43,44]. These crops are produced for direct household consumption, for trading at informal markets and in some cases the establishment of farmer cooperative schemes which sell the bulk of their grain to commercial markets. In South Africa, an estimated four million people engage in smallholder agriculture [45], with maize being the major crop and staple food in the country. Maize-based products are consumed by 67 to 83% of South Africans, and the mean consumption per day in rural areas is estimated between 476 and 690 g per person [46]. Maize produced in these areas is often affected by pre- and postharvest damage, of which fungal infection is one of the most significant constraints. Many subsistence farmers plant their fields with untreated seed reserved from the previous season [47], thereby increasing the risk of crop infection by fungi such as *F. verticillioides* [48]. Planting late in the season, maize monoculture and crop residues left on the soil surface further exacerbate the incidence of pests and fungal disease in the subsequent season [49]. Some farmers in the rural areas do not control insect pests such as stalk borer, yet insect pests contribute greatly to the spread of fungal infections and mycotoxin contamination [50].

Farmers are supported with inputs such as the supply and encouragement to use transgenic *Bt* maize hybrids to control stalk borers, and the prudent use of pesticides and fungicides. This enhances crop and food security, as well as providing for better mycotoxin control [51]. In addition, rural development initiatives encourage farmers to enter mainstream markets, which require high quality produce and contribute positively to mycotoxin control. This is supported by the national objectives for rural development in South Africa, which seek to address market inefficiencies within the subsistence agriculture sector [52] and include

- Formalising the rural market
- Increasing agricultural outputs from subsistence areas
- Encouraging agroprocessing operations within rural areas
- Enabling and providing linkages between producers and processors
- Promoting exports

## 3. Occurrence of Mycotoxins in Subsistence Crops in Southern Africa

Mycotoxins of concern in foodstuffs in low income countries are aflatoxin, fumonisin, ochratoxin A, zearalenone (ZEA), deoxynivalenol (DON) and several trichothecenes produced by certain *Aspergillus*, *Fusarium* and *Penicillium* species [6,53]. Acute mycotoxicoses have been reported in Africa and prolonged exposure to subacute levels of various mycotoxins is a risk factor for various human diseases, including cancer and childhood stunting [54,55]. Low individual income in the countries of the region contribute to high food insecurity and undiversified diets, predisposing populations to the consumption of mycotoxin contaminated foodstuffs [6,53,56]. Many of the agricultural crop regions in southern Africa are typified by hot, dry and sometimes humid conditions, erratic rainfall and frequent drought, providing an ideal environment for toxigenic fungi to thrive [57]. The inadequate drying of crops and intermittent drought spells in the region may necessitate food storage for long periods, thereby allowing for increased insect infestation, fungal proliferation and mycotoxin production [58]. Rural storage facilities frequently have no pest control, poor aeration, and poor moisture and temperature control.

Aflatoxins and fumonisins are widespread in major dietary and export targeted crops in southern Africa, with fewer cases of DON and patulin contamination [41,42,43,59]. Since most of the staples are also used as cash crops, the highest quality produce is often sold at local markets to commercial food processors or exported, leaving the lower quality food for home consumption and traditional beer brewing [58].

Mycotoxin occurrence has been reported in maize, peanuts, barley products, wheat, apple juice and in milk in South Africa, but limited reports exist of mycotoxin occurrence in agricultural products in other southern African countries, such as Botswana, Malawi, Mozambique, Zambia and Zimbabwe [60]. There is also little emphasis on legislating maximum levels in foodstuffs and even when such legislation exists, the capacity to enforce these measures is frequently lacking [61].

In rural South Africa, typical of many low income countries, food security is of primary concern and often outweighs food safety concerns [62]. The provinces of Limpopo (LP), Mpumalanga (MP), KwaZulu-Natal (KZN) and the EC have been shown to have high levels of fumonisin and/or aflatoxins during high risk crop seasons [39,47,63]. Most data on fumonisin natural occurrence in subsistence farming areas have been generated from traditional farming areas in the EC [1,37] and from northern KZN, where genetically modified maize was introduced to combat insect pests and control weeds [51]. Fumonisins and DON have been mainly detected in subsistence maize from the rural EC, while aflatoxin has been observed mainly in rural LP and KZN [62].

## 4. The Impact of Varying Climate Conditions on Contamination of Crops with Mycotoxigenic Fungi and Mycotoxins

Africa is one of the most vulnerable continents to climate variability and change because of multiple stresses and low adaptive capacity. The agriculture-based economy in Africa, including South Africa, is often directly linked to climatic conditions. By 2020, yields from rainfed agriculture could be substantially reduced by up to 50% in certain areas, which would further adversely affect food security and exacerbate malnutrition [64,65]. Climate conditions may change markedly with atmospheric CO_2_ concentrations expected to rise, and together with other greenhouse gases could result in increased global temperatures. Elevated CO_2_ levels and interactions with temperature and water availability may be conducive to the growth of mycotoxigenic fungal species. In South Africa, the likelihood of increased temperature is greater towards the interior, and less in coastal areas. Assuming a moderate to high increase in greenhouse gas concentrations by 2050, the coast is likely to warm around 1 °C and the interior around 3 °C. Drier conditions are predicted for the south west of the country in both seasons. Rainfall intensity (flooding) is likely to increase in the interior regions, but does not imply an increase in total rainfall. Greater evaporation rates are likely to increase drought incidence and intensity. There is reason to believe that these climate change factors can severely affect infection of grain crops with changing profiles of mycotoxigenic fungi and mycotoxin contamination. Tropical climates with high temperatures and longer periods of drought stress would have a significant impact on mycotoxin contamination and the amount of crop produced, directly impacting food security and nutritional quality.

Several predictive models for determining the levels of risk of mycotoxins in cereals are being developed [9,65,66]. Three-dimensional statistical models link fungal counts, mycotoxin production levels, molecular and geostatistic data to understand the impact that climate change may have on contamination of crops. System level models on the other hand, define the components of the life cycle of *F. verticillioides*, i.e., germination rates, growth rates, sporulation rates and fumonisin production rates in relation to climatic conditions, such as temperature and water availability, and the development phase of the host plant.

## 5. Mycotoxin Contamination and Risk Assessment

Modern food safety systems are designed around scientific principles that make them transparent, systematic and participatory. The overarching concept is one of risk analysis to ensure the availability of safe food and it is envisaged as being composed of three interlocking areas, namely risk assessment, risk management and risk communication [67]. In turn risk assessment is composed of hazard identification and hazard characterisation (previously referred to collectively as hazard assessment), exposure assessment and risk characterisation. All these elements of risk analysis can be applied to food safety in rural subsistence farming communities, but their implementation can differ from the approaches that are used in fully developed market economies. These risk analysis components are well recognised internationally and are jointly addressed by the FAO and the WHO. JECFA is the international risk assessment body and the CAC is the risk management body in which contaminant issues are addressed by the Codex Committee on Contaminants in Food (CCCF). The CCCF deals with various risk management concepts, primarily setting MLs and developing generalised codes of practice for prevention and reduction of specific contaminants. However, the MLs developed at CCCF and approved by CAC, although based on risk assessment outcomes, must also facilitate international trade by not impacting on world food supplies and have no relevance in small subsistence farming communities.

In the first instance, since food is both produced and mostly consumed on the same farm, risk communication must be addressed with suitable messages for the rural society. However, in market economies the presence of a food distribution chain allows for communication and intervention at multiple points from farm to household to the degree that the actual householders themselves need have only limited knowledge of potential chemical contaminants. In the same vein, risk management in rural areas needs to be directed at the farm/household level or at most, at small village rural markets, whereas the food value chain in developed markets allows management practices to be diversified along the distribution system. For these reasons, the implementation of regulations such as the setting of MLs for individual contaminants can be applied in these latter systems but have no validity in subsistence farming communities where food grown is locally consumed. The occurrence in certain areas of food insecurity between harvests further complicates the introduction of management systems which might impact food availability.

The communication and management strategies in subsistence farming communities are reliant on a comprehensive risk assessment applied to the particular villages being considered. It is important to recognise the universal nature of the outcome of JECFA hazard characterisation for contaminants such as mycotoxins. These characterisations are generally expressed as PMTDIs and are applicable across all population groups, both urban and rural subsistence. However, the exposure assessment part of risk assessment is both personal and individual and is influenced by the contaminant levels in the household food supply and individual consumption patterns. With some knowledge of these parameters, exposure assessments of varying complexity can be performed. Comparison of actual exposure (contaminant intake or PDI) versus PMTDI produces the risk characterisation and hence informs the risk managers of the nature of contaminants that need to be addressed and the degree to which this exposure may be influencing health outcomes. Authorities would then be able to calibrate their response based on the severity of the contaminant risk. Specific food intakes such as maize consumption in many African rural communities are extremely high, thus producing excessive exposures at modest contaminant levels [37,38]. For this reason, the contaminant level at which risk managers should react based on the risk characterisation may be very different from the international MLs set for trade by CAC.

## 6. Mycotoxin Dietary Exposure in Rural Maize Subsistence Farming Communities

Exposure assessment is accomplished by measuring or estimating the magnitude, frequency and duration of exposure to a hazard. It relies on utilising appropriate models such as a deterministic or a probabilistic approach [67,68]. The choice of an appropriate model is governed by numerous factors such as (i) type of assessment, (ii) dietary source of exposure, (iii) population demographics and socioeconomic characteristics, (iv) temporal aspects of the exposure, (v) data characteristics and availability, (vi) toxin characteristics, (vii) availability of resources and infrastructure and (viii) methodological uncertainties and variabilities [68,69,70,71].

Mycotoxin risk assessments in rural areas of the EC are mostly based on a deterministic approach due to the type of data available and access to resources and infrastructure. For this purpose, individual PDIs are calculated based on the multiplication of a fixed contamination level measured in the relevant food commodity (µg kg^−1^) by the amount of maize individually consumed (g day^−1^) and divided by body weight (kg). The mycotoxin of interest has remained FB based on the continued presence of high levels in home-grown maize. The first crude exposure assessment for rural subsistence farmers in the EC using estimated dietary maize intakes and a small data base of contamination levels was performed by Thiel et al. [72] and suggested PDIs of 14 µg kg^−1^ bw day^−1^ for those consuming “healthy” maize and 440 µg kg^−1^ bw day^−1^ if “mouldy” maize was to be consumed. Thereafter, a study using weighed food records of uncooked maize consumed over a 24-h period (24-h recall method) stratified by three different age groups (1–9, 10–17, and 18–65 years of age) was conducted in Bizana and Centane [37]. This is the first study of its kind that included rural children in mycotoxin risk assessment. Consumption data showed maize intakes (in g day^−1^) of 246 (1–9 years) and 368 (10–17 years) mostly similar for both areas, whereas the adults had an intake of 379 (Bizana) and 456 (Centane), respectively. Mean total FB (FB_1_ + FB_2_) levels of 1142 µg kg^−1^ (Centane) and 542 µg kg^−1^ (Bizana) in good maize were determined. The resultant PDIs (in µg kg^−1^ bw day^−1^) for children residing in Centane were 14 (1–9 years) and 8 (10–17 years) and those for Bizana included PDIs of 7 (1–9 years) and 4 (10–17 years), respectively. For adults assuming a body weight of 60 kg, the PDIs were 3 and 9 µg kg^−1^ bw day^−1^ for Bizana and Centane, respectively. With the development and validation of a culturally appropriate dietary assessment method aimed at estimating maize intake more in-depth exposure assessment became possible [73,74]. This method consists of a food frequency questionnaire (FFQ) with a series of food photographs with traditional maize-based dishes in different portions and ratios (for mixed dishes). This method provided an opportunity to distinguish between those consuming home-grown (mean of 474 g day^−1^), commercial (344 g day^−1^) and a mixture of the two (462 g day^−1^). With the assessment of individual body weights and the relevant total FB levels in home-grown maize (1142 µg kg^−1^), commercial maize (222 µg kg^−1^) and traditional Xhosa maize-based bear (369 ng mL^−1^), the PDIs were determined. In summary, the group consuming home grown maize had a higher PDI of 9 μg kg^−1^ bw day^−1^ compared to the 1 μg kg^−1^ bw day^−1^ of the group consuming commercial maize. The mixed consumption group showed a unique exposure profile when separating their individual PDIs into three, the combined PDI, PDI based on home-grown maize intake and the PDI for commercial maize consumption. Despite the lower contamination of commercial maize, those consuming higher amounts were exposed to fumonisin above the PMTDI. Furthermore, FB exposure based on intake of traditional maize-based beer [75] was stratified according to the frequency of consumption—once a month, once a week and 2–7 days a week—since beer drinking is not a daily occurrence. The respective mean PDIs ranged between 7 to 12 μg kg^−1^ bw per drinking event [76].

The accuracy of assessing mycotoxin exposure in rural parts of the EC has improved with the development and use of more standardised assessment methods to determine dietary intakes as well as contamination levels. The use of more reliable dietary assessment methods such as the 24-h recall/weighed record mix method or a validated maize-FFQ with food photograph series, produced similar PDIs for rural adults home-grown maize consumers in two different studies, that of 9 μg kg^−1^ bw day^−1^ [37,74,76]. Although the determinist approach is limited to providing a point in time estimate, it remains straightforward and easy to interpret. Moreover, in the context of a resource-limited setting where detailed national food consumption surveys and continued mycotoxin surveillance among high-risk areas are lacking, this approach is warranted.

## 7. Simulation Models to Assess Mycotoxin Exposure

**Figure 1:**
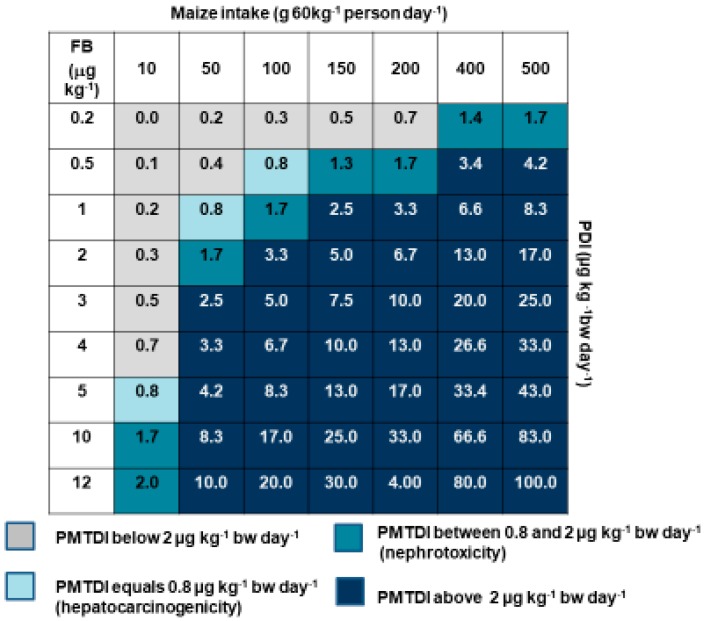
Interactive model illustrating the association between fumonisin B (FB) contamination levels (vertical column) and maize intakes (horizontal column) (adapted from Marasas et al. [1]).

**Figure 2:**
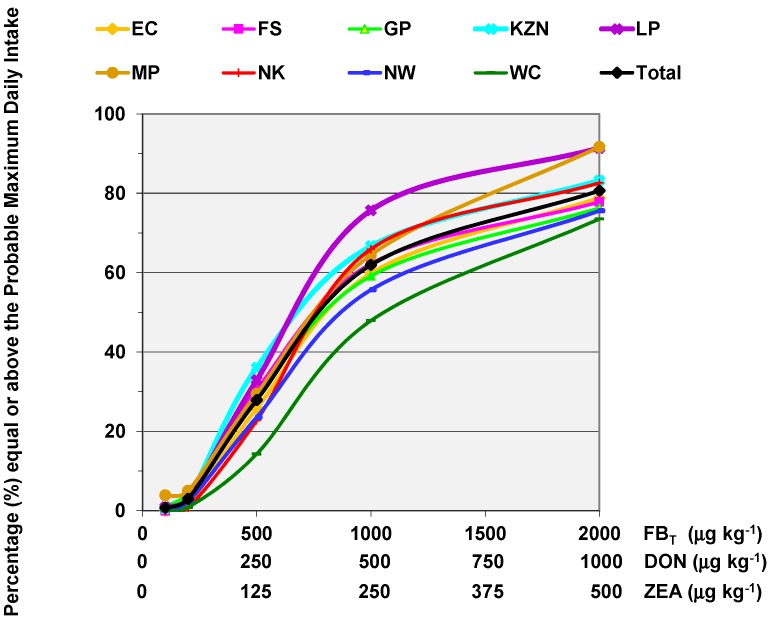
Mycotoxin Risk Assessment Model (MYCORAM) for total fumonisin B (FB_T_), deoxynivalenol (DON) and zearalenone (ZEA): Percentages of the South African maize consumers stratified by province equal or above the respective mycotoxin provisional maximum tolerable dietary intake (PMTDI) (adapted from Burger et al. [46], 2014, Oxford University Press). EC: Eastern Cape; FS: Free State; GP: Gauteng; KZN: KwaZulu-Natal; LP: Limpopo; MP: Mpumalanga; NC: Northern Cape; NW: North West; WC: Western Cape.

The first model of this kind was developed by Gelderblom et al. [77] and revised by Gelderblom and Marasas [78] (Figure 1). This model links arbitrary FB levels ranging from 0.2 to 12 µg kg^−1^, with maize intakes, 10 to 500 g day^−1^, using a general body weight of 60 kg. Different PDIs were calculated and colour-coded according to four FB risk assessment categories, i.e., PMTDI below or above 2 µg kg^−1^ bw day^−1^; PMTDI associated with nephrotoxicity (0.8 µg kg^−1^ bw day^−1^); and PMTDI associated with hepatocarcinogenicity (between 0.8 and 2 µg kg^−1^ bw day^−1^). This enabled the simulation of risk according to different maize intake patterns related to low- and high-income countries, as well as FB levels relevant to different sources of maize, i.e., home-grown, commercial or imported. However useful this model may be, it does not represent real-time maize intakes or body weight data. A study conducted in collaboration with a prominent South African grain-based manufacturing company, in the form of a national cross-sectional study, provided a unique opportunity to collect relevant sociodemographic information, maize dietary intakes and body weights of commercial maize consumers across South Africa [46]. Using this data and building on the previous model, the Mycotoxin Risk Assessment Model (MYCORAM) was developed for three relevant mycotoxins FB, DON and ZEA. Arbitrary levels of the three mycotoxins were plotted against specific maize intake categories (g kg^−1^ body weight day^−1^) and estimates the percentage of maize consumers exposed above the PMTDI for each mycotoxin (Figure 2). With the additional sociodemographic data that was available [46], this model was stratified by provincial region (*n* = 9). The purpose of this model was to calculate maximum levels to protect South African maize consumers. Using total FB, DON and ZEA levels in two commercial maize milling products (SUPER and SPECIAL maize meal) with overall lower levels compared to home-grown maize, the percentage of people exposed above the respective PMTDI, was dependent on the individual maize intake levels, body weight and subgroups variability related to the sampling strategy. Therefore, subgroups, such as men, known to consume more than women, and their risk may be masked within the MYCORAM. Stratifying risk according to the nine South African Provinces showed between 0.1 to 3% of the consumers residing in MP, LP, Gauteng and Kwa-Zulu Natal were above the limit for the three mycotoxins. Determining the relevant PDIs obtained using the same data, exposure ranged between 4 to 87 ng kg^−1^ bw day^−1^ which suggest that there is no risk. However, the MYCORAM is sensitive enough to identify those who are at risk and is therefore effective in predicting ML levels. Interesting when using FB levels relevant to home-grown maize, the percentage of consumers above the PMTDI ranged between 2 and 78% stratified by Province. Although the MYCORAM is unique and sensitive, it is only based on the intake patterns of commercial maize consumers and will require regular and expensive national surveys to remain relevant. In contrast, rural maize subsistence consumers remain the most vulnerable group in South Africa, and with the challenges of epidemiological surveys and food analyses a more valid, affordable and accessible approach is warranted, such as the application of biomarkers of exposure.

## 8. Biomarkers of Exposure

Studies on the human health impact of mycotoxins and the efficacy of intervention strategies require measurement of individual exposure, which is a function of both the intake of contaminated food and the degree of contamination, both of which are subject to analytical difficulties. With respect to the subsistence farming communities of southern Africa, this primarily means assessing maize consumption and fumonisin contamination. Given the preponderance of maize-based dishes, a culturally-specific Ratio and Portion Size Photo (RAPP) tool was developed and validated to aid in the measurement of maize intakes in the EC of South Africa [73,74]. Nevertheless, all traditional means of dietary assessment such as food frequency and 24-h dietary recall are subject to known recall biases. The assessment of the actual fumonisin contamination level in the prepared food for individual intakes is hampered by the nonhomogenous distribution of mycotoxins in maize samples. This adds uncertainty to individual exposure assessments. A further complication is the fate of fumonisins during the preparation of individual food dishes and the presence of modified mycotoxins, which are not detected by conventional mycotoxin analysis and which may or may not be bioavailable. Interindividual variations in absorption and possible metabolism of the contaminant further hamper correlations between exposure and adverse health outcomes. For these reasons researchers have sought a biomarker of exposure, which in a single measurement can indicate individual exposure to a contaminant [79]. Should such a measurement involve the physiological change induced by the mycotoxin, such a biomarker of physiological effect would be a powerful indicator for correlation with negative health effects.

Given the role of fumonisins as disruptors of sphingoid base metabolism, the first attempts at finding a biomarker involved measurements of sphingoid bases (sphinganine and sphingosine) in urine and plasma [31,38]. These were unsuccessful [80] and were supplanted by measurements of fumonisin itself in faeces, hair and urine [38]. Of these, urinary analysis of FB_1_ was shown to correlate well with tortilla consumption in a Mexican population [81]. A subsequent study in the rural maize farming area of Centane, EC, South Africa showed a positive correlation between urinary FB_1_ and fumonisin exposure, as measured by individual intake of contaminated maize. This urinary FB_1_ biomarker was also used to validate an intervention study based on sorting and washing of maize kernels prior to the cooking of a traditional maize-based porridge [82]. A further biomarker study has also shown the presence of urinary DON (including its glucuronide metabolites) and ZEA (including its zearalenol metabolites) in this rural population [83]. Future intervention studies should be designed to measure all three mycotoxins in urine as a measure of efficacy of the intervention protocol.

The future of mycotoxin exposure assessment including successful risk assessment, characterisation and management in rural maize farming areas in the EC will need continued surveillance using urinary multiple mycotoxin biomarker determinations. This method requires the noninvasive collection of urine samples by any trained fieldworker and does not require the collection of staple crops. Importantly, mycotoxin exposure should also include vulnerable groups such as infants and young children, known to receive maize porridge as a complementary food [73,74].

## 9. Intervention Models

Intervention models for reduction of fumonisins in maize are a worldwide priority focussing on the production chain and application preharvest, postharvest and during processing [84]. Physical and chemical control methods that are applied and have been commercialised involve sorting and flotation, solvent extraction, chemical detoxification by alkalisation, oxidation, irradiation and pyrolysis [85,86]. However, there are several concerns with regards to chemical methods, especially the potential health, safety and environmental risks [87,88,89]. As biological methods could have less impact on the nutritional value, quality, safety and sensory aspects of foods and less impact on the environment, there is a renewed interest in these methods as alternatives to chemical control methods.

### 9.1. Biologically Based Models for Control of Fusarium Growth and Fumonisin Production

Biological methods for mycotoxin reduction involve the application of natural resources, including plant material, microorganisms, DNA, RNA, proteins and phyllosilicate clay minerals [90]. Research performed in recent years demonstrated effective reduction of *Fusarium* infection and fumonisin production pre- and postharvest following the development of resistant and transgenic maize cultivars, treatment of maize involving phyllosilicate clay minerals, antioxidants, essential oils and phenolic compounds extracted from plant material, a variety of microbial species and detoxifying enzymes. Several of these methods have been commercialised for application alone, in combination or as part of an integrated control strategy.

#### 9.1.1. Resistant and Transgenic Maize Cultivars

Preharvest approaches involve the development of transgenic maize hybrids and the further development of natural resistance in maize through plant breeding. Various plant biotechnological techniques are used to minimise the incidence of pests and mycotoxin-producing fungi, and accomplish degradation of mycotoxins in planta [91,92,93]. These approaches require extensive genomic resources and knowledge on the biochemical and regulatory pathways during biosynthesis of mycotoxins and plant–pathogen interactions, i.e., genetic maps, genome sequences, expression sequence tag (EST) libraries and integrated gene indexes [90,92,94,95]. Transcriptional changes associated with *F. verticillioides* infection in resistant and susceptible maize genotypes was studied with next-generation RNA sequencing, which provided information on genetic markers involved in recognition, signalling and host resistance mechanisms, and valuable interpretations on defence responses. Mapping of chromosomal regions encoding resistance to Fusarium ear rot as quantitative trait loci (QTL) and the employment of marker-assisted QTL are also valuable tools being developed for maize hybrid development. Another approach involves the expression of catabolic enzymes in planta to detoxify mycotoxins before they accumulate in the plant [91].

In order to further develop natural resistance, the expression profiles of maize genes and associated proteins during fungal infection in susceptible vs. resistant genotypes, provide valuable information. Infection with *F. verticillioides* resulted in the upregulation of genes encoding a range of proteins related to cell rescue, defence and virulence [93]. In resistant maize lines, defence-related genes were transcribed at high levels before infection and provided defence against the fungus, while in susceptible maize lines, the defence genes were induced, though not adequately enough to prevent further development of the disease. Resistance and susceptibility is underpinned by plant–pathogen interactions. During fungal infection, the plant carbohydrate metabolism is affected by induced invertase and sucrose synthase enzyme activities and the formation of hexoses which are required for fungal growth. Maize lipoxygenase derived oxylipins (e.g., jasmonic acid), are also known for regulating plant defence against pathogens.

#### 9.1.2. Transgenic *Bt* Maize

Insect-resistant, transgenic (*Bt*) maize, expressing insecticidal Cry proteins derived from *Bacillus thuringiensis*, effectively reduce certain types of lepidopteran and other insect infestations, culminating in both lower *Fusarium* infection and fumonisin production [96]. *Bt* protected maize and food derived from *Bt* crops have been certified safe for humans, animals and the environment by the World Health Organisation (WHO) and the US Environmental Protection Agency (EPA), as well as several other regulatory agencies throughout the world. Extensive field trials have confirmed frequently lower fumonisin concentrations in *Bt* maize hybrids [97], with the annual benefits in the USA estimated at $23 million [98].

*Bt* maize has seen impressive implementation rates since its introduction into South Africa in the 1998/1999 crop season [99]. Field trials conducted in 2002 and 2003 at two locations in a commercial maize-growing area in the North West Province of South Africa, showed that the fumonisin levels in the *Bt* hybrids were between 39 and 83% lower than their respective non-*Bt* isolines [100]. Results from a study conducted in rural areas in northern KZN from 2004–2008 [51], demonstrated a clear advantage of *Bt* maize over traditional, landrace seed and conventional commercial hybrids, with *Bt* exhibiting 40% less fumonisin contamination than the traditional varieties. In addition, relative to their non-*Bt* commercial hybrids, *Bt* maize also exhibited on average 16% less fumonisin contamination. In South African rural areas, farmers are more likely to purchase herbicide tolerant transgenics than *Bt* hybrids due to the advantage of effective weed control with glyphosates each season, compared to sporadic stalk borer infestations which do not warrant the use of *Bt* hybrid seed [101]. In some areas, farmers have been supplied with starter transgenic seed packs for one or two seasons, but are then expected to purchase the seed in the following years. These farmers prefer to revert back to their open pollinated or traditional varieties which they can grow from year to year, without purchasing expensive commercial seed each season [102].

#### 9.1.3. Biocontrol Microorganisms

Control of *F. verticillioides* infection and fumonisin production preharvest in maize has been demonstrated by biocontrol bacterial species including *Bacillus mojavensis* [90]; lactic acid bacterial strains, e.g., *Pediococcus pentosaceus* L006 [103]; *Trichoderma viride* [104]; andrhizobacterial isolates of *Arthrobacter globiformis*, *Azotobacter armeniacus*, *Pseudomonas solanacearum* and *Bacillus subtilis* [105]. Reduction of fungal growth and fumonisin production is accomplished during interaction between the host plant, plant pathogen and biocontrol microorganisms, underpinned by competition for nutrients and space, parasitism of the pathogen, secretion of antifungal compounds, induction of systemic resistance, biofilm formation and reactive oxygen species in defence responses [85,87]. *Bacillus subtilis*, *T. viride* and *P. pentosaceus* are generally regarded as safe (GRAS) by the United States Food and Drug Administration (US FDA) [106]. *Bacillus subtilis* RRC101 effectively reduces fumonisin accumulation during the endophytic growth phase of *F. verticillioides* (= *F. moniliforme*) in maize [107], while *T. viride*, known for its wide range of extracellular lytic enzymes, is used in biological fertilisers for control of soil borne pathogenic fungi in crops. Interaction between and sensitivity of the biocontrol bacterial strain towards other microbial strains and mycotoxins present in the biological niche could, however, affect the efficacy of this approach.

#### 9.1.4. Essential Oils and Antioxidants

Many antioxidants, essential oils and phenolic compounds extracted from plant material exhibit resistance to fungal growth by inhibition of fungal enzymes which are required for growth [108]. These plant derived compounds are especially favourable for reduction of fungal growth and associated mycotoxins during storage. The inhibitory effect in planta is generally achieved with higher concentrations than obtained in vitro, because of matrix interference. High concentrations of these compounds could, however, affect the organoleptic properties of maize. The antioxidants butylated hydroxyanisole (BHA) and propylparaben (PP) reduce *F. verticillioides* and *F. proliferatum* growth, and fumonisin production at a variety of water activities and incubation temperatures in vitro [109]. Treatment with BHA and PP resulted in a significant (*p* < 0.001) reduction in fungal hydrolytic enzyme activity and combination treatments resulted in greater reduction [110,111]. BHA and PP have GRAS status and are permitted by the US FDA to be used as antimicrobial agents in food.

#### 9.1.5. Phyllosilicate Clay Minerals

Phyllosilicate clay minerals, such as montmorillonite, adsorb irreversibly to mycotoxins through cation-exchange. Montmorillonite has high adsorption ability in comparison with other phyllosilicates due to its large molecular structure and surface area that increases considerably when wet. Incorporation of montmorillonite during food and beverage processing resulted in effective detoxification of aflatoxin and fumonisin contaminated food and aqueous solutions [112,113,114,115]. Incorporation into animal feed decreases the bioavailability and associated toxicities of these mycotoxins in the gastrointestinal tract of animals. Because several montmorillonite clays have GRAS status, they could be applied effectively and economically in food and feed industries [86]. High levels are, however, often required for inclusion in animal feed, while the interaction with food- and gut-based nutrients remains unclear and the possibility of accumulation of dioxin remains a concern. Application in impoverished communities which are nutritionally compromised also needs to be further investigated.

#### 9.1.6. Enzymatic Degradation

Enzymatic degradation of mycotoxins in food sources postharvest is a new research field with plenty of scope for novel developments and improvement of the safety aspects of treatment methods [90,101]. Targeted modification of the chemical structures by enzymatic cleavage or conversion of chemical bonds/groups associated with the toxicity of the mycotoxin has been the focus of many research approaches [116,117,118,119,120,121,122,123,124]. The toxicological effects of the fumonisins have been centred on the free amino group, while the tricarballylic acid moiety would appear to be a requirement for the effective absorption from the gut [116]. Microorganisms and enzymes capable of degrading FB_1_ include carboxylesterase and amino oxidase enzymes of *Exophiala spinifera* ATCC 74269, *Rhinocladiella atrovirens* ATCC 74270 as well as carboxylesterase and aminotransferase enzymes of Bacterium ATCC 55552 and *Sphingopyxis macrogoltabida* MTA144 [121,125,126,127,128,129]. De-esterification of FB_1_ and subsequent deamination of hydrolysed FB_1_ (HFB_1_) with the formation of 2-keto HFB_1_ has been shown to be an effective detoxification approach. Recently, a commercial fumonisin esterase FumD (EC 3.1.1.87), FUM*zyme*^®^ (BIOMIN, Austria) of bacterial origin (*S. macrogoltabida* MTA144), capable of effectively hydrolysing FB_1_ was developed. HFB_1,_ exhibited a far reduced toxicity in the gut of pigs when considering changes to intestinal morphology; intestinal immune response; modulation of the sphinganine/sphingosine ratio in the liver and plasma; while it lacks any hepatotoxicity [128]. FUM*zyme*^®^ has been registered safe for humans, animals and the environment by the European Food Safety Authority (EFSA). Although enzymatic FB detoxification has become a promising approach, and it is currently successfully applied in the animal feed industry [129].

### 9.2. Community-Based Intervention Models

In rural subsistence farming areas, culturally sensitive, practical, and cost-effective biologically based methods of reduction at community level are relevant [101]. Effective reduction of fumonisins in maize has been demonstrated with hand-sorting, winnowing, flotation, washing, dehulling of maize kernels and combinations thereof in vitro and in field studies, and was recently reviewed [90]. These methods are applicable to the preparation of maize-based food in rural subsistence farming households. In many African countries, such as South Africa, Benin, Nigeria, Tanzania and Malawi, crops are customarily hand-sorted prior to storage and cooking [130,131,132,133]. With regards to hand-sorting, flotation and dehulling of maize, hand-sorting proved the most effective for populations with limited food resources. Hand-sorting of maize kernels results in 69–71% reduction in fumonisin levels and results in much lower mass loss than obtained with dehulling [131,132,133,134,135,136]. Hand-sorting is only effective, however, if the sorted mouldy maize is discarded and not used for animal feed and beer making. Mechanical shelling and dehulling of maize by various methods (shelling by hand; handle-operated shellers; motorised shellers) result in 57–65% reduction in fumonisin levels; however, it causes damage to maize kernels and considerable mass loss [134,136,137,138]. Following sorting, the washing of maize with water results in additional 13-15% reduction in fumonisin levels [82,134,136]. In South Africa a practical and culturally sensitive hand-sorting and washing intervention method was developed and evaluated for reduction of fumonisin exposure in a rural subsistence maize farming community. The two-step maize kernel water wash intervention method developed by Van der Westhuizen et al. [82,133,139] involved visually sorting of maize kernels followed by a 10 min water wash method in a rural subsistence maize farming community in the EC of South Africa, resulting in an overall decrease of 84% in fumonisins; 62% reduction in the PDI; and 52% reduction in urinary excretion of FB_1_.

## 10. Conclusions

Maximum regulatory levels set by the CAC for mycotoxins in maize are aimed at safeguarding world food supplies, while still facilitating international trading. The MLs are intended to be health protective such that mycotoxin exposures will be below the PMTDI set by JECFA. These regulations, however, have no validity in rural subsistence farming communities in southern Africa where food is grown and locally consumed. Maize is consumed in high quantities resulting in excessive exposure to mycotoxins, even though the mycotoxin contamination levels are modest. In low income countries, country-specific data on exposure to mycotoxins are lacking, therefore comprehensive risk assessments should be performed in rural areas by employing the available statistical models and dietary assessment methods to quantify exposure, and authorities should calibrate their response based on these results.

It is clear that the reduction of fumonisin exposure in rural subsistence farming communities requires an integrated approach and cannot be achieved purely by regulatory means. Detailed understanding of agricultural practices, mycotoxin occurrence, climate change, human exposure and risk are warranted to guide adequate intervention programmes. Intervention models should be implemented to perform risk communication and create awareness in communities at risk. It is, furthermore, imperative that intervention methods for reduction of mycotoxins in maize should be implemented. A range of biologically based products for control of mycotoxigenic fungi and mycotoxins pre- and postharvest in maize have been developed and commercialised, including the introduction of biocontrol microorganisms, enzymes, montmorillonite clay adsorbents, as well as antioxidants, essential oils and phenolic compounds extracted from plants. Application of these methods is, however, limited in rural subsistence farming communities due to a lack of infrastructure, resources and access to technologies. As a result, the WHO [67] made recommendations for reduction of mycotoxins in staple grains applicable to rural subsistence farming communities. Community-based practical and integrated interventions are relevant and need to be implemented, such as hand-sorting, flotation, washing, winnowing, shelling, dehulling and milling of maize kernels and/or combinations thereof. Integration of technological methods with community-based approaches should be encouraged, to further enhance reduced exposure in these communities. Challenges regarding the sustainability of these strategies include food security, customary uses for mouldy maize and traditional beliefs and practices [55,76,134]. Public health interventions should be culturally sensitive; be implemented through educational campaigns; and must have financial and infrastructural support to be achievable.
